# Long-Standing Rheumatoid Arthritis With Elevated Epstein–Barr Virus IgG Titers: A Case Report From India

**DOI:** 10.7759/cureus.93117

**Published:** 2025-09-24

**Authors:** Gabriela Fernandes, Suraj Palavilayil, Meshael Shaikh

**Affiliations:** 1 Department of Periodontics and Endodontics and Oral Biology, School of Dental Medicine, State University of New York Buffalo, Buffalo, USA; 2 Department of Medical Education, Blackpool Teaching Hospitals NHS Foundation Trust, London, GBR; 3 Rheumatology, Manipal Hospitals, Bangalore, IND

**Keywords:** autoimmunity, case report, epstein–barr virus, rheumatoid arthritis, rheumatology

## Abstract

Rheumatoid arthritis (RA) is a systemic autoimmune disease with multifactorial etiology, and Epstein-Barr virus (EBV) has been proposed as an environmental trigger contributing to RA pathogenesis. Previous studies in India have shown that sera from early RA patients react to deiminated proteins encoded by EBV, with antibodies such as anti-viral citrullinated peptide (anti-VCP) and anti-EBNA-1 present exclusively in RA patients, suggesting a role for EBV in inducing disease-specific antibodies. Globally, RA patients demonstrate elevated antibodies against multiple EBV proteins, including VCA, EA, EBNA-1, and EBNA-2, independent of disease duration, activity, or treatment, and possess increased numbers of circulating EBV-infected B cells. Experimental and clinical evidence indicates that these antibodies, particularly anti-citrullinated collagen type II (CII) antibodies, may contribute to immune self-perpetuation and joint pathology. We report a 62-year-old woman with a 20-year history of seropositive RA, clinically stable on methotrexate and tofacitinib, who exhibited persistently elevated EBV viral capsid antigen (VCA) IgG titers (>750 U/mL), with asymptomatic trace proteinuria and microscopic haematuria. No RA flare or EBV reactivation was observed. This case provides region-specific evidence from India that complements prior studies, illustrating that EBV seropositivity persists in long-standing RA and may continue to contribute to disease-specific humoral responses. These findings underscore the need for longitudinal studies and advanced viral assays to clarify the role of EBV-related markers in RA pathogenesis.

## Introduction

Rheumatoid arthritis (RA) is a chronic autoimmune disease characterized by persistent synovial inflammation, progressive joint damage, and multisystem involvement. Despite advances in therapeutics, including disease-modifying antirheumatic drugs (DMARDs), its precise etiology remains incompletely understood, and patients often require lifelong management [1]. Epstein-Barr virus (EBV) has long been investigated as a potential environmental contributor to RA. Early studies by Yao et al. (1986) demonstrated impaired viral control and higher circulating EBV loads in RA patients, while Costenbader and Karlson (2006) reported that, despite near-universal EBV exposure, RA patients exhibit consistently elevated antibody titers and viral loads [2,3]. Trier et al. (2018) identified antibodies to citrullinated EBV-derived peptides almost exclusively in RA patients, highlighting disease specificity and illustrating viral-host molecular mimicry, whereby viral proteins resemble host antigens and trigger autoimmunity [4]. Magnusson et al. (2010) found that EBV detection in bone marrow predicted response to rituximab, suggesting its potential as a biomarker [5].

Data from the Indian subcontinent are limited, with only one study by Deo et al. investigating EBV-related antibody responses in early RA patients. Deo et al. demonstrated that sera from these patients reacted to deiminated proteins encoded by EBV, including viral citrullinated peptide (VCP) and EBNA-1, suggesting that EBV may contribute to the induction of disease-specific antibodies in Indian RA patients [6].

We present a long-standing RA patient from India with persistently elevated EBV IgG titers. The aim is to complement existing Indian data on viral contributions to RA pathogenesis.

## Case presentation

A 62-year-old female with a 20-year history of seropositive RA had been managed with methotrexate for two decades and tofacitinib for the past two years. Her surgical history included a unilateral hip replacement in 2010 and bilateral knee replacements in 2015 and 2017. She also had a two-year history of hypertension, which had previously been managed with amlodipine but was currently well controlled without medication. At routine follow-up, the patient reported improved general well-being and remained clinically stable, with no flare or exacerbation of joint pain.

On physical examination, her blood pressure was 122/78 mmHg without antihypertensive medication, pulse 78/min, and temperature afebrile. No acute synovitis was observed, although residual deformities of the hands and knees consistent with long-standing RA were present. Chest, cardiac, and abdominal examinations were unremarkable.

Her illness had begun at age 42 with symmetrical small joint arthritis, later progressing to involve large joints. She denied smoking or alcohol consumption, and there was no family history of autoimmune disease.

Laboratory investigations in July 2025 showed a complete blood count within normal limits, erythrocyte sedimentation rate (20 mm/hr), and C-reactive protein (1.63 mg/L). Renal and liver function tests were unremarkable. Urinalysis demonstrated trace proteinuria and microscopic haematuria (22-24 RBC/hpf), which were asymptomatic and managed conservatively. In August 2025, EBV serology was performed to explore the longstanding hypothesis of EBV involvement in RA, as prior international and Indian studies, including Deo et al., have reported higher EBV antibody titers in RA patients compared to controls. Results revealed markedly elevated EBV VCA IgG titers (>750 U/mL; reference <20) and positive EBNA IgG. Early antigen IgG and EBV DNA PCR were not performed. The patient remained clinically stable and continued her existing DMARD regimen without modification. These results are summarized in Table 1.

**Table 1:** Laboratory results (July–August 2025). CBC: complete blood count; ESR: erythrocyte sedimentation rate; CRP: C-reactive protein; ALT: alanine aminotransferase; EBV: Epstein–Barr virus; VCA: viral capsid antigen

Test	Result	Reference range	Comment
Haemoglobin	13.4 g/dL	11.5–15.5	Normal
WBC count	8010/µL	4000–11,000	Normal
Platelets	263,000/µL	150,000–455,000	Normal
ESR	20 mm/hr	0–20	Upper normal
CRP	1.63 mg/L	<5.0	Normal
Creatinine	0.87 mg/dL	0.7–1.2	Normal
ALT (SGPT)	28 U/L	5–35	Normal
Fasting glucose	110 mg/dL	70–110	Borderline high
Post-prandial glucose	121 mg/dL	80–140	Normal
Urinalysis	Trace protein, 22–24 RBC/hpf	—	Microscopic haematuria
EBV VCA IgG (Aug 2025)	>750 U/mL	<20 negative; ≥20 positive	Markedly elevated

## Discussion

Research has increasingly explored the link between RA and elevated serum immunoglobulin G (IgG) antibodies, particularly those directed against the EBV envelope capsid antigen [7,8]. This association is of particular interest because it may provide important clues about the immunological pathways involved in the onset and progression of RA. RA is a chronic autoimmune disease marked by persistent inflammation of the joints, which can cause pain, swelling, and progressive joint destruction. While the exact cause of RA remains uncertain, evidence suggests that viral infections, especially EBV, could be a contributing factor [7-10]. 

Clinical investigations have shown that people with RA often display significantly higher levels of IgG antibodies against EBV capsid antigens compared to healthy individuals [8]. One study reported that the rate of IgA/VCA antibody positivity was considerably greater in RA patients than in those with systemic lupus erythematosus (SLE) or in control groups, reinforcing the possibility that EBV infection is closely tied to RA development. Prospective studies have revealed that individuals with raised IgG antibodies targeting EBV early antigens are at a higher risk of developing RA. This elevation has been linked to increased serum IgM rheumatoid factor levels, an immune marker commonly associated with the disease [8-10]. 

Global studies have demonstrated higher EBV antibody titers and viral loads in RA patients, and antibodies to citrullinated EBV peptides have been identified almost exclusively in RA, suggesting viral-host molecular mimicry [7,8]. In India, data are limited, with only one study by Deo et al. investigating early RA patients [6]. Deo et al. observed that sera from these patients reacted to deiminated proteins encoded by EBV, including viral citrullinated peptide (VCP) and EBNA-1, highlighting a possible role of EBV in inducing disease-specific antibodies. Our case extends this observation to a patient with long-standing RA, illustrating that elevated EBV VCA IgG titers persist beyond early disease stages and providing additional, region-specific evidence from the Indian subcontinent, where RA burden is substantial but viral associations remain underreported.

The patient’s incidental urinalysis abnormalities were mild, asymptomatic, and likely unrelated to EBV, possibly reflecting age or long-term DMARD use. While VCA IgG elevation alone is not diagnostic and does not indicate viral reactivation, the markedly elevated titers align with patterns reported in other RA populations. Limitations include the absence of EBV DNA quantification, viral load monitoring, and early antigen testing, which could have clarified viral activity; such assays will be incorporated in future observational studies.

## Conclusions

This case of long-standing RA with persistently elevated EBV VCA IgG titers emphasizes the need for cautious interpretation of viral serology. It complements the only existing Indian study and contributes regional data to the global understanding of EBV-RA associations. Future studies with longitudinal follow-up, viral load monitoring, and advanced assays are needed to determine whether EBV markers may have mechanistic or biomarker relevance in RA pathogenesis.

## References

[1] Zhang X, Li B, Liu Y, Jiang M (1999). Clinical study on antibodies against EBV in sera of patients with rheumatoid arthritis [Article in Chinese]. Zhongguo Yi Xue Ke Xue Yuan Xue Bao.

[2] Yao QY, Rickinson AB, Gaston JS, Epstein MA (1986). Disturbance of the Epstein-Barr virus-host balance in rheumatoid arthritis patients: a quantitative study. Clin Exp Immunol.

[3] Costenbader KH, Karlson EW (2006). Epstein-Barr virus and rheumatoid arthritis: is there a link?. Arthritis Res Ther.

[4] Trier NH, Holm BE, Heiden J (2018). Antibodies to a strain-specific citrullinated Epstein-Barr virus peptide diagnoses rheumatoid arthritis. Sci Rep.

[5] Magnusson M, Brisslert M, Zendjanchi K, Lindh M, Bokarewa MI (2010). Epstein-Barr virus in bone marrow of rheumatoid arthritis patients predicts response to rituximab treatment. Rheumatology (Oxford).

[6] Deo SS, Shetty RR, Mistry KJ, Chogle AR (2010). Detection of viral citrullinated peptide antibodies directed against EBV or VCP: in early rheumatoid arthritis patients of Indian origin. J Lab Physicians.

[7] Catalano MA, Carson DA, Slovin SF, Richman DD, Vaughan JH (1979). Antibodies to Epstein-Barr virus-determined antigens in normal subjects and in patients with seropositive rheumatoid arthritis. Proc Natl Acad Sci U S A.

[8] Westergaard MW, Draborg AH, Troelsen L, Jacobsen S, Houen G (2015). Isotypes of Epstein-Barr virus antibodies in rheumatoid arthritis: association with rheumatoid factors and citrulline-dependent antibodies. Biomed Res Int.

[9] Miljanovic D, Cirkovic A, Jermic I (2023). Markers of Epstein-Barr virus infection in association with the onset and poor control of rheumatoid arthritis: a prospective cohort study. Microorganisms.

[10] Fechtner S, Berens H, Bemis E (2022). Antibody responses to Epstein-Barr virus in the preclinical period of rheumatoid arthritis suggest the presence of increased viral reactivation cycles. Arthritis Rheumatol.

